# Boldine as a neuroprotective agent against motor neuron degeneration in models of amyotrophic lateral sclerosis

**DOI:** 10.3389/fncel.2025.1640590

**Published:** 2025-09-16

**Authors:** Carlos A. Toro, Wei Zhao, Patricio Garcia Silva, Daniela Retamal-Santibáñez, Fabiola Rojas, Jiangping Pan, Nicholas Johnson, Yorley Duarte, Christopher P. Cardozo, Juan C. Sáez, Brigitte van Zundert

**Affiliations:** 1 Spinal Cord Damage Research Center, James J. Peters VA Medical Center, Bronx, NY, United States; 2 Department of Medicine, Icahn School of Medicine at Mount Sinai, New York, NY, United States; 3 The Bronx Veterans Medical Research Foundation, Bronx, NY, United States; 4 Faculty of Medicine and Faculty of Life Sciences, Institute of Biomedical Sciences (ICB), Universidad Andres Bello, Santiago, Chile; 5 Facultad de Ciencias de la Vida, Center for Bioinformatics and Integrative Biology (CBIB), Universidad Andrés Bello, Santiago, Chile; 6 Instituto de Neurociencias, Centro Interdisciplinario de Neurociencias de Valparaíso, Universidad de Valparaíso, Valparaíso, Chile; 7 Millennium Nucleus of Neuroepigenetics and Plasticity (EpiNeuro), Santiago, Chile; 8 Department of Neurology, University of Massachusetts Chan Medical School (UMMS), Worcester, MA, United States

**Keywords:** boldine, ALS, connexin hemichannel blockage, neuroprotection, motoneuron

## Abstract

Amyotrophic lateral sclerosis (ALS) is a progressive neurodegenerative disease characterized by motor neuron loss. Current FDA-approved treatments offer only modest benefits. Connexins (Cx), proteins that mediate intercellular communication have emerged as potential therapeutic targets, with increased Cx hemichannel (HC) activity observed in ALS models, and blocking Cx HC activity prevents motor neuron loss *in vitro*. Boldine, a natural compound with both Cx HC-blocking and antioxidant properties, has shown neuroprotective potential. This study investigated boldine’s effects in ALS models. *In vitro*, spinal cord cell cultures exposed to conditioned media from mutant SOD1^G93A^ astrocytes showed a 50% reduction in motor neuron survival, elevated Cx HC activity, and increased reactive oxygen species (ROS). Boldine treatment significantly reduced Cx HC activity and ROS, and increased motor neuron viability. *In vivo*, oral boldine was well-tolerated in male mutant SOD1^G93A^ mice starting at 7 weeks of age. Mice receiving 50 mg/kg/day showed a median survival increase of 9 days (132 vs. 123 days), though not statistically significant. Functional assessments revealed delayed disease progression: in the horizontal ladder rung walk test, boldine-treated mice exhibited a 36.8% reduction in crossing time and 21.2% fewer stepping errors. Improved scores were also observed on the Basso Mouse Scale at later stages, indicating preserved locomotor function. However, boldine had no significant effect in the rotarod test. These results support boldine’s neuroprotective effects in ALS, particularly in fine motor coordination and locomotor performance. Its reduction of Cx HC activity and oxidative stress highlights boldine’s promise as a potential therapeutic candidate for ALS.

## Introduction

Amyotrophic lateral sclerosis (ALS) is a progressive neuromuscular disease characterized by the degeneration of upper motor neurons in the motor cortex and brainstem and lower motor neurons located in spinal cord. Symptoms of the disease typically include dysphagia, dysarthria, limb muscle weakness with fasciculations, and respiratory difficulty (McDermott and Shaw, 2008). Following symptom onset, ALS patients have a median survival of 2–5 years, with respiratory failure being the primary cause of mortality (Yoshida et al., 1986). To date, the United States Food and Drug Administration (FDA) has approved only two treatments for ALS: riluzole, which reduces glutamate excitotoxicity, and edaravone, a free radical scavenger. These drugs modestly extend survival by approximately 3 and 6 months, respectively, underscoring the need for more effective therapies (Abe et al., 2014; Bensimon et al., 1994; Jaiswal, 2019).

ALS can be classified as sporadic ALS (sALS), accounting for 90% of cases, and familial ALS (fALS), which constitutes 10% of cases and arises from inherited genetic mutations. Risk factors for sALS include smoking, environmental exposures, physical trauma, and military service (Talbott et al., 2016). In fALS, several genetic mutations have been implicated, with mutations of the C9ORF72 and SOD1 genes being the most prevalent. C9ORF72 mutations account for approximately 40% of fALS cases and involve repeat expansions whose physiological roles remain under investigation (Farg et al., 2014). Mutations in the SOD1 gene, which encodes superoxide dismutase 1, contribute to 20% of fALS cases. These mutations often lead to a toxic gain-of-function rather than a loss of enzymatic activity (Saccon et al., 2013).

The development of ALS pathology is multifactorial, with motor neuron degeneration being influenced by neighboring cells, emphasizing the non-cell-autonomous nature of the disease (Ilieva et al., 2009; Van Harten et al., 2021). Based on studies *in vitro* with cultures and *in vivo* with mouse models, it is widely accepted that motor neuron degeneration in ALS involves non-cell-autonomous mechanisms, through interactions between various cell types such as microglia, astrocytes, oligodendrocytes, mast cells, and muscle cells (Ilieva et al., 2009; Van Harten et al., 2021; Stoklund Dittlau and Van Den Bosch, 2023; Harcha et al., 2021; Garcés et al., 2024; Martínez et al., 2024). Particularly, and using astrocyte-conditioned media (ACM) from mutated astrocytes, there is compelling evidence that ALS astrocytes (excessively) release soluble factors that induce non-cell-autonomous toxicity in motor neurons. Soluble factors implicated in ALS include: (1) glutamate, (2) oxygen and nitrogen reactive species (ROS/RNS), (3) ATP (4), cytokines/chemokines, (5) mutant SOD1, and (6) inorganic polyphosphates (polyP) (Stoklund Dittlau and Van Den Bosch, 2023; Harcha et al., 2021; Garcés et al., 2024; Arredondo et al., 2022).

Experimental models, particularly transgenic mice overexpressing the human SOD1^G93A^ mutation, have provided critical insights into ALS mechanisms. As of today, the high-copy-number SOD1^G93A^ transgenic mouse model (termed hereafter mutSOD1) remains a cornerstone of ALS research because this model closely recapitulates the human histopathological and clinical symptoms of ALS, including motor neuron death and muscle atrophy, and exhibit a stable and well-established disease progression, enabling preclinical evaluation of gene and pharmacological therapies (Gurney et al., 1994; van Zundert and Brown, 2017). *In vivo* and *in vitro* studies (using co-cultures and ACM) have also highlighted motor neuron hyperexcitability, characterized by persistent sodium ion influx, mitochondrial dysfunction, and oxidative stress, leading to apoptosis (Garcés et al., 2024; van Zundert et al., 2012). Importantly, these effects can be mitigated by antioxidants or sodium channel blockers (Stoklund Dittlau and Van Den Bosch, 2023; Fritz et al., 2013; Rojas et al., 2015).

Recent evidence implicates connexin hemichannels (Cx HCs) in ALS pathogenesis. These HC, composed of six connexin subunits, mediate intercellular communication and regulate small-molecule exchange. Dysregulated expression of Cx HCs in ALS models has been associated with motor neuron degeneration (Almad et al., 2016; Cui et al., 2014; Lagos-Cabré et al., 2017; Almad et al., 2022). Notably, pharmacological inhibition of Cx HCs *in vitro* and genetic ablation of Cx43 *in vivo* improves motor neuron survival in mutSOD1 ALS models, highlighting their potential as therapeutic targets (Almad et al., 2016; Almad et al., 2022).

Cx HCs, particularly those composed of connexin 43 (Cx43), have emerged as critical contributors to ALS pathogenesis and promising therapeutic targets. Cx HCs are hexameric membrane channels that mediate intercellular communication by regulating the exchange of ions and small molecules. Under physiological conditions, these channels present low activity, which increases transiently to support normal signaling. However, in ALS, astrocytes and microglia exhibit dysregulated and upregulated Cx43 hemichannel activity, leading to the aberrant release of neurotoxic factors such as ATP, glutamate, and ROS, (Almad et al., 2016; Cui et al., 2014; Lagos-Cabré et al., 2017; Almad et al., 2022; Orellana et al., 2011). This glial-mediated toxicity contributes to motor neuron degeneration and disease progression. *In vivo* studies using the SOD1^G93A^ mouse model have shown that astrocyte-specific deletion of Cx43 delays disease onset, preserves motor neuron function, and extends survival (Almad et al., 2022). Additional studies confirmed that Cx43 expression and hemichannel activity are also elevated in human ALS tissue and in patient induced pluripotent stem cell (iPSC) derived astrocytes, implicating this mechanism in both fALS and sALS (Almad et al., 2016; Almad et al., 2022). Moreover, pharmacological blockade of Cx HCs with specific inhibitors such as Gap19 or tonabersat leads to neuroprotection *in vitro* and *in vivo* (Almad et al., 2016; Almad et al., 2022). Together, these findings highlight Cx HCs as mechanistically relevant and tractable targets for ALS intervention.

Boldine, a natural alkaloid with potent antioxidant properties, has emerged as a promising Cx HC blocker (Saez et al., 2024). Beyond its ability to scavenge reactive oxygen and nitrogen species (ROS/RNS), boldine directly inhibits Cx HCs (Hernández-Salinas et al., 2013), preventing the toxic extracellular release of signaling molecules. This dual mechanism positions boldine as a compelling candidate for mitigating ALS pathology by reducing oxidative stress and hemichannel activity.

Based on this, we hypothesized that boldine could enhance motor neuron survival by blocking Cx HCs and reducing oxidative stress. Our objective was to evaluate the protective effects of boldine on motor neuron survival and function using *in vitro* and *in vivo* mutSOD1 ALS model.

## Methods

### Animals

Those experiments with transgenic mouse models of ALS were approved by the Institutional Animal Care and Use Committee at James J. Peters Veterans Affairs Medical Center (JJP VAMC) IACUC #CAR-20-11. We used hemizygous transgenics mice harboring the human mutation SOD1^G93A^ (High number of copies; mutSOD1) obtained from Laboratories Jackson (Cat. No. 0022726, Bar Harbor, ME, USA). Non-transgenic littermates (NTg) were used as controls. The presence of the transgene was identified by end-point PCR (Fritz et al., 2013).

### Spinal cord cultures

Sprague–Dawley rats at 14 days of gestation were anesthetized in a CO₂ chamber, their 14-day embryos (E14) were extracted, and ventral spinal cord cultures were prepared as previously described (Arredondo et al., 2022; Fritz et al., 2013; Rojas et al., 2014). Briefly, the ventral spinal cords of these embryos were dissected, mechanically dissociated, and enzymatically digested with 0.25% trypsin (Gibco) for 20 min at 37°C. After digestion, cells were transferred to a 15 mL Falcon tube containing 10 mL of a nutrient medium composed of 70% MEM (Invitrogen), 25% Neurobasal Medium (Invitrogen), 1% N2 supplement (Invitrogen), 1% penicillin–streptomycin (Gibco), 2% horse serum (Hyclone), and 1 mM pyruvate (Sigma Aldrich). The cells were centrifuged at 1,000 rpm for 2 min, and the pellet was resuspended in 2 mL of nutrient medium. The suspension was mechanically dissociated using flame-polished Pasteur pipettes. Cells were then counted and seeded at a density of 4 × 10^6^ cells/mL in 24-well plates previously coated with 1 mg/mL poly-L-lysine (Sigma). Prior to seeding, the cells were supplemented with E18 chicken leg extract at a ratio of 0.6 μL extract per 1 mL of nutrient medium.

### Preparation of astrocyte conditioned media (ACM) from mutSOD1 mice

Astrocyte conditioned media (ACM) was prepared as previously described by our laboratory (Arredondo et al., 2022; Fritz et al., 2013; Rojas et al., 2014). Astrocyte cultures were derived from the spinal cords of postnatal day 1 or 2 (P1–P4) mutSOD1 or non-transgenic littermates (NTg) mice as controls. Cultures were maintained in DMEM (Hyclone) supplemented with 10% fetal bovine serum (Hyclone) and 1% penicillin–streptomycin (Invitrogen) at 37°C with 5% CO₂. Once cultures reached ~95% confluence (approximately 2 weeks), they were shaken at 200 rpm for 6 h in an incubator to remove microglial cells. The medium was then replaced with the nutrient medium used for spinal cord cultures and incubated for 7 days. The conditioned medium was then collected, supplemented with 4.5 mg/mL D-glucose and 0.6 μL/mL chicken muscle extract, filtered, and diluted to 0.1X based on previous reports from our laboratory.

### Treatment of spinal cord cultures with boldine and DMSO

Four concentrations of boldine (Härting Company)—100 μM, 50 μM, 25 μM, and 12.5 μM—were tested alongside corresponding dimethyl sulfoxide (DMSO, Sigma Aldrich) vehicle controls at equivalent dilutions (0.1, 0.05, 0.025, and 0.0125%). Each concentration was independently added to VSCDC cultures at 4 days after initial plating for culture and incubated for 3 days at 37°C and 5% CO₂. At 7 days *in vitro*, cells were fixed with 4% paraformaldehyde (PFA, Sigma) for 20 min, followed by immunofluorescence staining and motor neuron survival assessment.

### Immunofluorescence in spinal cord cultures

Immunofluorescent staining to access motor neuron viability in spinal cord cultures was conducted as described previously (Van Harten et al., 2021; Arredondo et al., 2022; Fritz et al., 2013; Rojas et al., 2014; Urushitani et al., 2006; Nagai et al., 2007; Mishra et al., 2020). PFA fixed cells were washed with 1X PBS (Winkler), permeabilized with 0.5% Triton X-100 (Sigma) for 20 min and blocked with goat serum (Invitrogen) for 30 min. Cells were incubated overnight at 4°C with primary antibodies: mouse anti-SMI32 (1:600, Abcam, ab187374), which recognizes non-phosphorylated neurofilaments in motor neurons, and rabbit anti-MAP2 (1:300, Invitrogen, OSM00030W), which recognizes microtubule-associated protein 2 in neurons and interneurons; SMI-32/MAP2-identified motor neurons in spinal cord cultures are also positive for choline acetyltransferase (ChAT) (Arredondo et al., 2022). Secondary antibodies (Alexa Fluor 488 goat anti-mouse, 1:500, Invitrogen; Alexa Fluor 546 goat anti-rabbit, 1:500, Invitrogen) were applied for 2.5 h at room temperature. After three 15-min PBS washes, cells were mounted using a nuclear-staining mounting medium (Prolong Antifade Mountant, ThermoFisher). Images were acquired with a Nikon Eclipse Ti-U epifluorescence microscope equipped with a 14-bit camera and mercury lamp.

Astrocyte cultures were fixed with 4% PFA, washed with 1X PBS, permeabilized with 0.5% Triton X-100 for 20 min, blocked with goat serum for 30 min and immunoassayed for Cx43 by incubating cells with a primary antibody against Cx43 (1:200; Invitrogen; Cat. #13-8300). Antibody binding was visualized with the appropriate fluorescent secondary antibody, Alexa Fluor 488 Goat anti-mouse (1:500; Invitrogen; Cat. #A-11029). Immunolabeled astrocytes were documented on an inverted Nikon Eclipse Ti-U microscope equipped with a SPOT Pursuit™ USB Camera CCD (14-bit), Epi-FL illuminator, mercury lamp, and Sutter Smart-Shutter with a lambda SC controller. Cells were photographed using a 20× objective.

### Motor neuron survival analysis

Motor neuron survival was assessed from immunofluorescence images obtained with a 20× objective using the Nikon Eclipse Ti-U microscope. Survival percentage was calculated by dividing the number of motor neurons (SMI32+) by the total number of neurons (MAP2+) and normalizing the control condition to 100% (Arredondo et al., 2022; Fritz et al., 2013; Rojas et al., 2014). Ten fields per condition from three independent experimental sets were analyzed.

### Reactive oxygen species (ROS) production assay

Intracellular ROS levels were measured as previously described by our laboratory (Martínez et al., 2024; Rojas et al., 2015). Briefly, a stock of 5 mM of the CM-H2DCF-DA probe (Invitrogen, Cat. No. C6827) was prepared fresh in DMSO and then diluted in the culture medium to a final concentration of 1 μM. Cells were washed with PBS 1X to remove the different media conditions, and the CM-H2DCF-DA probe was applied for 30 min at 37°C in the dark. To facilitate the incorporation of the probe into cells, 0.004% pluronic acid F-127 (Invitrogen, Cat. No. P-3000MP) was added. After incubation, the probe CMH2DCF-DA dissolved in the culture media was removed, and cells were washed twice with PBS 1X to apply the culture medium to the spinal cord neurons. Imaging was made using an epifluorescence microscope (Nikon Eclipse Ti-U; objective 20X) and excitation and emission wave λex/λem = 492–495/517–527 nm. At least 3 fields were taken for each condition, and at least 10 cells per field were used for the quantification. The analysis of images was done using ImageJ software (NIH, Bethesda, MD, USA).

### *In vivo* experimental design and boldine administration

Male transgenic mutSOD1 mice (Jackson Laboratory, stock #002726) were used for this study. At 7 weeks of age, mice were randomly assigned to receive boldine (Millipore Sigma, Cat. No. B3916) or vehicle control (peanut oil) administered twice daily, as previously described (Toro et al., 2023). Boldine was delivered at 9:00 AM and 5:00 PM directly into the cages. To prepare the treatment, boldine was first dissolved in a solution of dimethyl sulfoxide (DMSO) and peanut oil and then incorporated into peanut butter (PB) to achieve a final dosage of 50 mg/kg body weight (BW) per day. Each dose was delivered in a 1.0 g PB bolus per mouse, with the final concentration of DMSO kept below 2%. Control animals received an identical PB formulation lacking boldine. All animals consumed the entire bolus within 1 h of administration and maintained complete consumption throughout the study period. To ensure consistent intake and reduce novelty-related stress, mice were habituated to both the peanut butter vehicle and behavioral testing equipment for 1 week prior to the start of treatment. Body weight was recorded prior to the first boldine administration and then monitored weekly thereafter.

### Ethidium uptake test

Ethidium bromide (EtBr) does not fluoresce, but it becomes fluorescent upon intercalation with nucleic acids serving as permeability probe to test the activity of hemichannels present in the cell membrane (Johnson et al., 2016). Thus, changes in membrane permeability through variation in Cx HC activity can be evaluated using the EtBr uptake assay. EtBr uptake was performed as described by (Schalper et al., 2008). In brief, spinal cord cultures were first exposed to the experimental conditions: Ctrl-medium, NTg-ACM, mutSOD1-ACM alone or in the presence of 25 μM boldine, or 200 μM La^3+^. Immediately afterwards, cells were treated with EtBr from a 25 mM stock solution in water (Sigma) and diluted to 5 μM final concentration and incubated at 37°C with 5% CO₂ for 30 min. After incubation, cultures were washed and fixed with 4% PFA for 20 min. Immunofluorescent staining was then performed to specifically label motor neurons (as indicated above), using a mouse anti-SMI32 primary antibody and an Alexa Fluor 488-conjugated goat anti-mouse secondary antibody. Following immunostaining, motor neurons were identified under a Nikon Eclipse Ti-U epifluorescence microscope using the 488 nm channel, while EtBr uptake was assessed in the 546 nm channel. Three independent experimental replicates were conducted. For each condition in each replicate, at least 10 motor neurons were analyzed. The red fluorescence intensity of EtBr was quantified in the soma of SMI32-positive motor neurons using ImageJ software. Fluorescence intensities were normalized to the average control condition, which was assigned a value of 1. The normalized values were used for comparative analysis across experimental groups.

### Determination of survival

Survival was defined as the number of days after birth that passed before the animal met any of the following conditions for euthanasia for humane reasons: no spontaneous breathing or movement for 60 s with no response to pain; the animal was unable to roll over to the normal position within 10 s following a gentle nudge; or complete hind limb paralysis (Zhao et al., 2012). Euthanasia was performed using carbon dioxide (CO₂) administered at a flow rate of 30–70% of the chamber volume per minute, for 5 min. No other chemical methods or tools were used.

### Evaluation of hindlimb function

Hindlimb function was determined weekly using the Basso Mouse Scale which provides a standard scoring system for hindlimb locomotor function (Basso et al., 2006). Mice were placed in the center of a 3-foot diameter open field and recorded using a GoPro camera. Recordings were used by blinded technicians to score hindlimb function on a 9-point scale where 9 is normal function and 0 represents no hindlimb movement.

### Evaluation of coordination

The horizonal ladder rung test was used as a measurement of overall coordination as previously described (Toro et al., 2023). The animal is allowed to cross a standard horizontal ladder with 73 rungs. The time needed to cross was recorded. The animals were recorded from underneath the ladder using a GoPro camera. Recordings are scored by blinded technicians and the total number of hind limb steps as well as the number of steps for which a hindlimb dragged over, slipped off or otherwise was not properly placed on a rung was determined.

### Rotarod testing

Mice were tested on an accelerating rotarod (7,650 Ugo Basile Biological Research Apparatus, Comerio, Italy) as previously described (Zhao et al., 2012). In brief, mice were placed onto a rotating grooved cylinder (facing away from the experimenter). The rotational speed began at 4 RPM and incrementally augmented to 40 RPM over 300 s or until the animal fell. The time at which the animal falls is defined as the latency and is recorded. If the animal has not fallen by 300 s, latency is recorded as 300 s. A diminishing latency indicates declining performance. A value of 0 s is suggestive of severe muscular weakness and impaired coordination. Mice were tested weekly, beginning at 50 days of age, until they could no longer perform the test. Before boldine administration and testing, mice underwent a one-week training period wherein they were introduced to the apparatus and handled by the experimenter daily. Testing was conducted during the last 4 h of the day portion of the light cycle in an environment with minimal stimuli such as noise, movement, or changes in light or temperature.

### Molecular docking and dynamics studies

Human Cx43 electron microscopy structure (PDB ID: 7F92) was used for the *in silico* experiments focusing on previously identified D4 and valproic acid binding site (Lee et al., 2023) Interaction between boldine and the binding site was evaluated with the Glide software (Halgren et al., 2004) in Schrödinger Suite (Release 2023-3, Schrödinger LLC, New York, NY) using the software’s default settings to ensure uniformity and reproducibility. The structure of boldine was obtained from PubChem in its two-dimensional form, and the LigPrep suite was used to prepare it for analysis. The docking analysis was conducted in standard precision mode, which provided a flexible sampling of the ligand conformations (Friesner et al., 2006). The Glide Docking Score algorithm was used to assess and score the produced poses, with its default settings (Schrödinger Release 2023-3: Glide, Schrödinger, LLC, New York, NY, 2023). The binding site was identified as druggable because of its affinity for interacting with the ligand, and conformations that showed the highest favourable docking scores were selected. Brief molecular dynamic simulation was performed using Desmond software from the Schrödinger suite. The Simulation Interaction Diagram tool of Schrödinger’s Maestro platform was used to analyse the generated molecular dynamic data. Subsequently, the binding free energy of the Cx43-boldine complexes was estimated using the Molecular Mechanics-Generalized Born Surface Area MM-GBSA approach (Schrödinger Release 2023-3: Glide, Schrödinger, LLC, New York, NY, 2023).

### Dye uptake assay in HeLa cells using real-time imaging

For these experiments, we followed a previously published protocol (Soleilhac et al., 2021). Briefly, HeLa cells were seeded at a density of 40,000 cells per well in 24-well tissue culture plates and maintained in Opti-MEM medium supplemented with 10% FBS. Cells were incubated at 37°C in a humidified atmosphere containing 5% CO₂ for 24 h prior to transfection. Cells were transfected with a plasmid encoding human Cx43 tagged with GFP (hCx43-GFP) using the jetOPTIMUS transfection reagent (Genesee Scientific), following the manufacturer’s instructions. Cells were incubated for 24–48 h post-transfection to allow for expression of the fusion protein. Cells were then washed gently with HBSS buffer (negative control) or calcium-free HBSS buffer. Cells were then incubated with TO-PRO-3 iodide (5 μM) alone or pre-treated with boldine (50 μM) for 2 min before the addition of TO-PRO-3 (5 μM). Live-cell imaging was performed using the EVOS FL Auto Imaging System (Thermo Fisher Scientific) with a 10 × objective. Time-lapse imaging was conducted in three channels: Phase contrast, Green fluorescence (GFP, to identify hCx43 expression) and Cyan fluorescence (TO-PRO-3). Images were acquired at 0 (baseline), and every 5, 10, 15, 20, 25, and 30 min following TO-PRO-3 addition. Fluorescence images were analyzed using Fiji/ImageJ software. TO-PRO-3–positive cells were identified in merged channel images using intensity thresholding. Quantitative analysis of dye uptake was performed by calculating the number of TO-PRO-3–positive cells over time, providing a measure of hemichannel activity and the effect of boldine treatment.

### Statistical analysis

All data are presented as mean ± standard error. Results from cell culture studies were from three or more independent experiments. For animal studies, the number of animals is indicated in figure legends. Statistical analyses were performed using one-way ANOVA followed by Tukey’s *post hoc* test. Differences were considered statistically significant at *p* < 0.05 (*), *p* < 0.01 (**), and *p* < 0.001 (***). GraphPad Prism 10 software was used for all statistical analyses. Survival curves were analyzed using Kaplan-Meir curves.

## Results

### Effect of boldine on motor neuron survival in spinal cord cultures

To assess the cytotoxicity of boldine on motor neurons in spinal cord cultures, we determined the effects of various concentrations of boldine and its vehicle, DMSO in cell viability. Previous studies reported that DMSO at 1% can induce mitochondrial dysfunction, oxidative stress, and membrane potential loss in astrocyte cultures (Yuan et al., 2014). Additionally, boldine has been applied *in vitro* at 100 μM in mouse cortical astrocyte cultures (Yi et al., 2017). Based on these findings, we designed a boldine concentration curve (100, 50, 25, and 12.5 μM) with corresponding DMSO controls (0.1, 0.05, 0.025, and 0.013%) to identify non-cytotoxic levels in spinal cord cultures (Supplementary Figure 1). For this, 4 days *in vitro* (DIV) spinal cord cultures were exposed to the boldine and vehicle concentrations and incubated for 3 days (Supplementary Figure 1A). At 7 DIV, cultures were fixed and double immunostained for MAP2 and for unphosphorylated neurofilament-H (SMI32) to identify motor neurons (SMI32^+^/MAP2^+^-positive cells) or interneurons (SMI32^−^/MAP2^+^- positive cells) (Arredondo et al., 2022; Fritz et al., 2013; Nagai et al., 2007; Mishra et al., 2020). Analysis revealed that 100 μM boldine was toxic, significantly reducing motor neuron survival by 50% compared to controls (cultures in basal medium termed Ctrl-medium) (Supplementary Figure 1B). However, boldine at 50, 25, and 12.5 μM showed no significant cytotoxicity, with highest motor neuron survival detected at 25 μM (~90%) with no detected toxicity with vehicle DMSO alone (Supplementary Figure 1B). Based on these findings, 25 μM boldine was selected for subsequent experiments.

### Hemichannel activity in wild-type spinal cord cultures exposed to mutSOD1-ACM and treated with boldine

Previous studies reported that motor neurons and glial cells of the spinal cord express connexins (Bautista et al., 2014). In ALS, the Maragakis group demonstrated significantly elevated Cx43 protein levels in cultured mutSOD1 mouse astrocytes, as well as in spinal cord tissue from end-stage NTg and mutSOD1 mice, as determined by western blot analysis (Almad et al., 2016). In agreement with these findings, our previous study—using the same culture conditions as in the present work—also showed, via western blot analysis, that Cx43 protein levels were significantly increased in mutSOD1 mouse astrocytes compared to control astrocytes from non-transgenic littermates (NTg) (Lagos-Cabré et al., 2017). Consistent with these findings, immunostaining assays (from one independent culture) also support elevated Cx43 levels in mutSOD1 astrocytes compared to NTg astrocytes (Supplementary Figure 2). These findings further support the link between SOD1 mutation, astrocyte-mediated Cx HC dysregulation, and motor neuron vulnerability.

**Figure 1:**
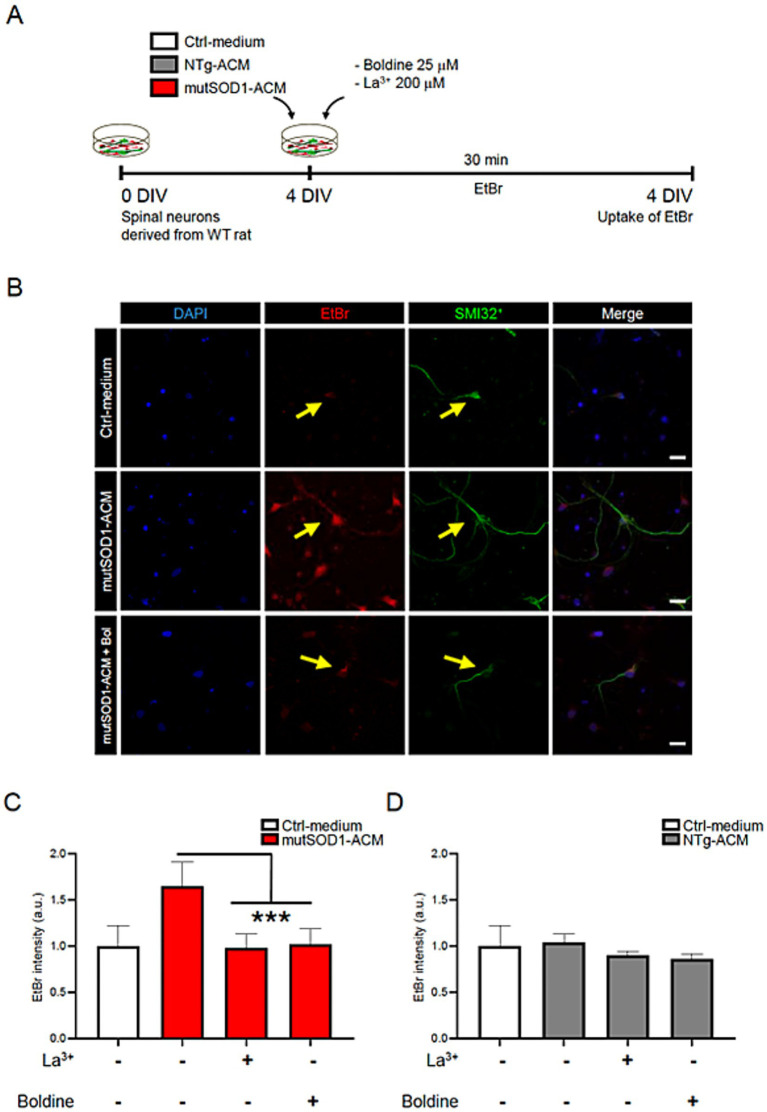
Boldine prevents Cx HC activity in motor neurons exposed to mutSOD1-ACM. **(A)** Experimental design: 4 DIV spinal cord cultures were incubated with EtBr (5 mM) alongside with Ctrl-medium, NTg-ACM and mutSOD1-ACM in the presence or absence of boldine (25 μM), or La^3+^ (200 μM), a Cx HC blocker and used as a positive control. Each condition was incubated for 30 min at 37°C with 5% CO₂, followed by 4% PFA fixation for immunofluorescence. **(B)** Representative fluorescence showing EtBr uptake (red) in SMI-positive motor neurons (green) when cultures are treated with mutSOD1-ACM, but not with Ctrl-medium or mutSOD1-ACM plus boldine. DAPI staining shows neuronal nuclei in different conditions. Scale bar: 50 μm. **(C,D)** Bar graphs show fluorescence intensity quantification of EtBr uptake in motor neurons after applying mutSOD1-ACM **(C)** and NTg-ACM **(D)** alone or with La^3+^ or boldine. Values represent the mean ± standard error of at least three independent experiments, analyzed via one-way ANOVA followed by Tukey’s post-hoc test. *** *p* < 0.001 vs. mutSOD1-ACM.

As ethidium bromide (EtBr) enters cells via active Cx HCs, we investigated whether boldine could similarly modulate Cx HC activity and hence EtBr uptake in spinal cord cultures exposed to mutSOD1-ACM (Yi et al., 2017). As controls, we used ACM derived from non-transgenic littermates (NTg-ACM) and control medium (Ctrl-medium). Specifically, 4 DIV spinal cord cultures were exposed to EtBr for 30 min at 37°C with mutSOD1-ACM, NTg-ACM or Ctrl-medium in the absence or presence of boldine (25 μM) (Figure 1A). SMI32 immunostaining was used to identify motor neurons, and EtBr fluorescence intensity was measured as an indicator of Cx HC activity. Cultures exposed to mutSOD1-ACM displayed significantly increased EtBr uptake in SMI32-postive motor neurons compared to controls (Figures 1B–D). Interestingly, co-incubation with boldine significantly reduced EtBr fluorescence to control levels. Lanthanum ion (La^3+^), a known Cx HC blocker (Contreras et al., 2002), also significantly reduced EtBr fluorescence. These results indicate that boldine effectively prevents mutSOD1-ACM induced Cx HC activity.

### Boldine reduces ROS/RNS accumulation in spinal cord cultures exposed to mutSOD1-ACM

**Figure 2:**
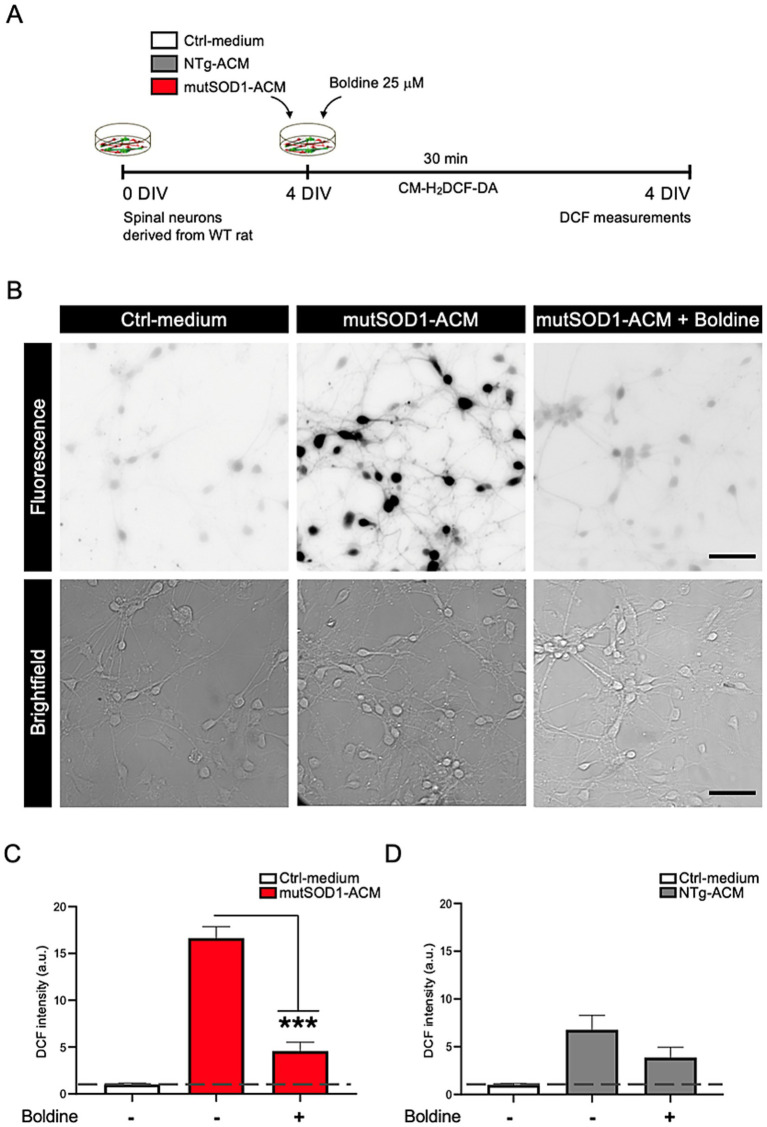
Boldine prevents increased intracellular ROS/RNS levels in spinal cord cultures exposed to mutSOD1-ACM. **(A)** Experimental design: 4 DIV spinal cord cultures were exposed to Ctrl-medium, NTg-ACM and mutSOD1-ACM alone or with boldine (25 μM) for 30 min, then washed and incubated with CM-H2DCF-DA for another 30 min. Changes in fluorescence due to ROS/RNS level increases were recorded and quantified. **(B)** Representative fluorescence (top panel) and bright-field (lower panel) images showing increased fluorescence signal when cultures were treated with mutSOD1-ACM, which was reduced in the presence of boldine to levels observed in Ctrl-medium treated cultures. Scale bar: 50 μm. **(C,D)** Bar graphs show ROS/RNS level quantifications based on CCF fluorescence intensity after application mutSOD1-ACM **(C)** and NTg-ACM **(D)** alone or with boldine (25 μM). Values represent the mean ± standard error of at least three independent experiments, analyzed via one-way ANOVA followed by Tukey’s post-hoc test. ∗∗∗*p* < 0.001 vs. mutSOD1-ACM.

To assess boldine’s antioxidant properties, we exposed spinal cultures to mutSOD1-ACM, NTg-ACM, or Ctrl-medium for 30 min, with or without boldine (25 μM) and assessed ROS/RNS using the CM-H_2_DCF-DA probe (Figure 2A). The CM-H_2_DCF-DA probe fluoresces upon reaction with intracellular ROS/RNS (Eruslanov and Kusmartsev, 2010). Application of mutSOD1-ACM to the spinal cultures significantly increased intracellular ROS levels, compared to Ctrl-medium and NTg-ACM, respectively (Figures 2B–D). Co-incubation of mutSOD1-ACM with boldine reduced intracellular ROS/RNS levels to those observed in NTg-ACM control conditions (Figures 2C,D). These results demonstrate boldine’s capacity to reduce intracellular ROS/RNS levels induced by mutSOD1-ACM.

### Protective effect of boldine on motor neuron survival exposed to mutSOD1-ACM

**Figure 3:**
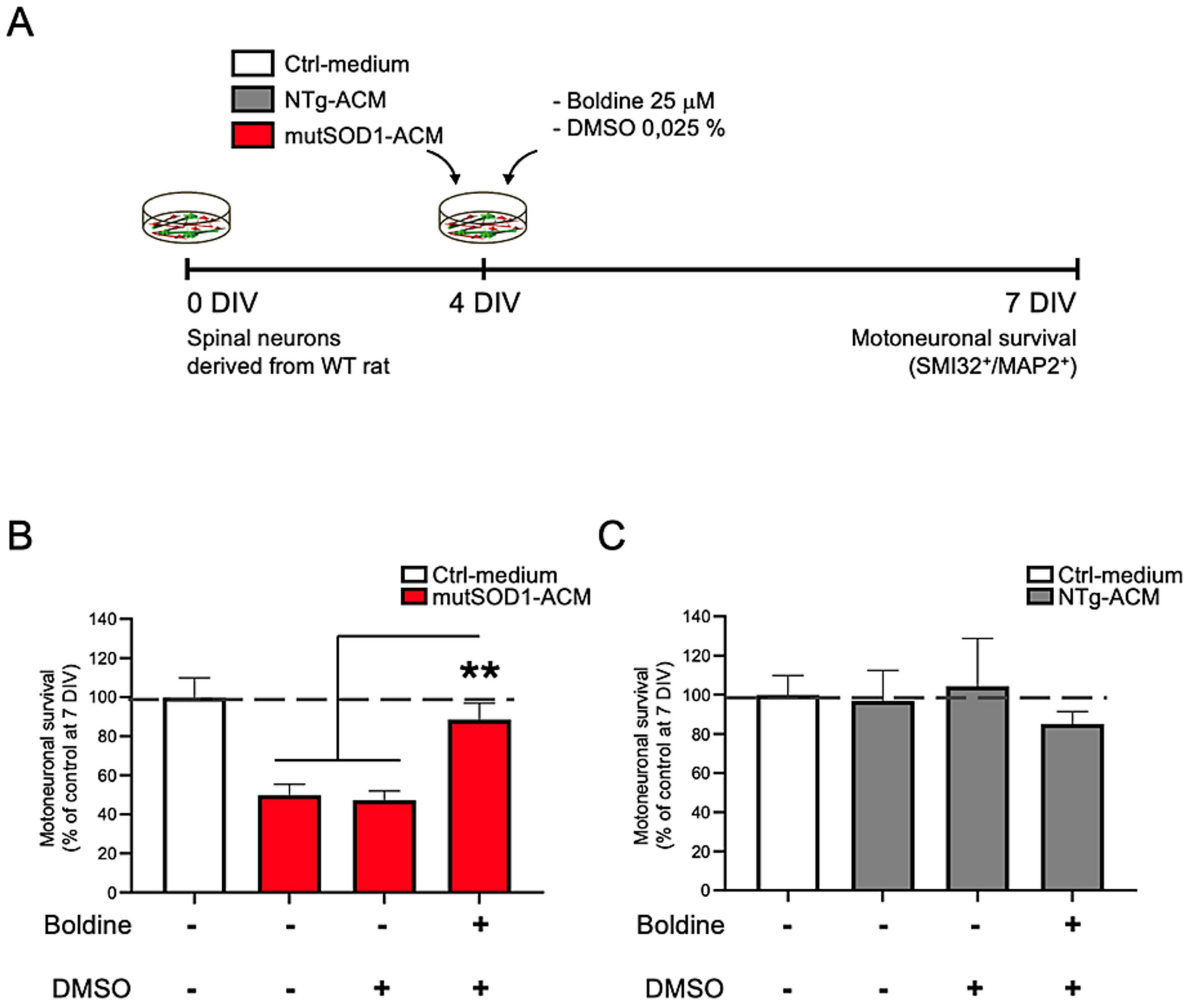
Boldine prevents motor neuron death in spinal cord cultures exposed to mutSOD1-ACM. **(A)** Experimental design: 4 DIV spinal cord cultures were exposed to Ctrl-medium, NTg-ACM and mutSOD1-ACM alone or with boldine (25 μM) for 3 days. At 7 DIV, cultures were fixed and motor neuron survival was analyzed via immunofluorescence assays (SMI32 and MAP2 staining). **(B,C)** Bar graphs show motor neuron survival quantification under mutSOD1-ACM **(B)** and NTg-ACM **(C)** conditions alone, with DMSO (0.025%), or with boldine (25 μM). Values represent the mean ± standard error of at least three independent experiments, analyzed via one-way ANOVA followed by Tukey’s post-hoc test. ∗∗*p* < 0.01 vs. mutSOD1-ACM with boldine and DMSO.

Exposure to mutSOD1-ACM is known to cause 50% motor neuron death in spinal cord cultures (Arredondo et al., 2022; Fritz et al., 2013; Rojas et al., 2014; Nagai et al., 2007; Mishra et al., 2020). To evaluate boldine’s neuroprotective effect, we exposed spinal cord cultures to mutSOD1-ACM, NTg-ACM, or Ctrl-medium in the absence of presence of boldine (25 μM) from 4 to 7 DIV, (Figure 3A). In agreement with previous studies, immunofluorescence analysis (SMI32 and MAP2 staining) and subsequent quantitative data confirmed that mutSOD1-ACM, but not NTg-ACM, reduced motor neuron survival by 50% (Figures 3B,C). Importantly, administration of boldine to mutSOD1-ACM significantly improved motor neuron survival, demonstrating its neuroprotective efficacy (Figure 3B).

### Boldine prolongs life span in male mutSOD1 mice

**Figure 4:**
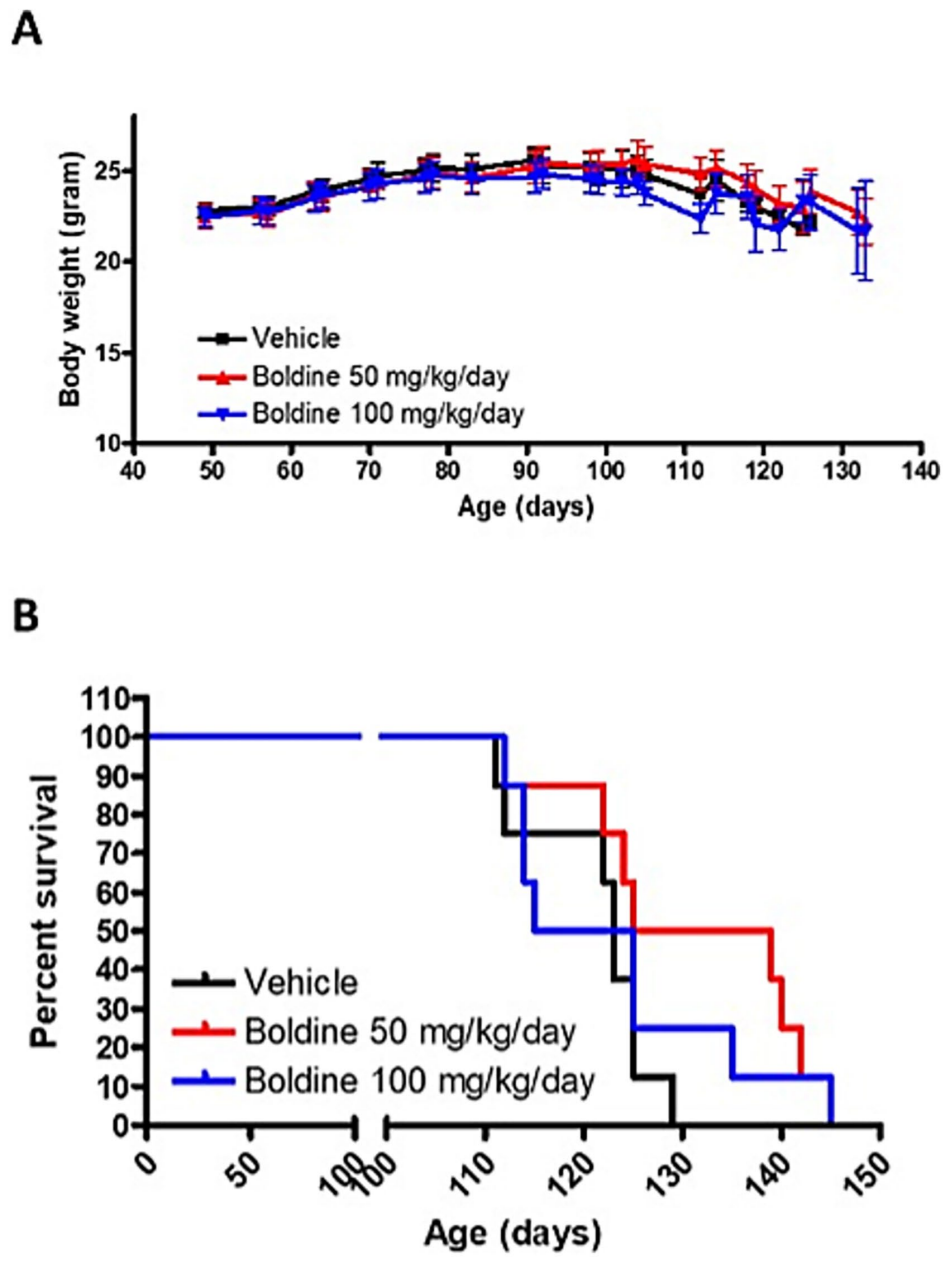
Effect of boldine on the survival of mutSOD1 mice. Male mutSOD1 mice were fed with vehicle, 50 mg/kg BW/day or 100 mg/kg BW/day boldine (*n* = 8 per group) starting from 7 weeks of age. **(A)** Body weight was measured weekly. **(B)** Kaplan–Meier analysis comparing boldine-treated mice to litter matched vehicle-treated control mice. Mice were euthanized when they met the humane endpoint. *N* = 8 per group.

We conducted a study to test the tolerance of boldine in mutSOD1 mice and its potential to slow down disease progression and/or increase survival. Male mutSOD1 mice were treated with 2 doses of boldine through daily oral delivery starting from 7 weeks of age. There was no significant difference of body weight between vehicle-treated group and boldine-treated group at any time point (Figure 4A), suggesting that oral administration of boldine at 50 mg/kg/day and 100 mg/kg/day are well tolerated in mutSOD1 mice. Encouragingly, mice that received 50 mg/kg/day showed a median survival of 132 days, which is 9 days longer than the vehicle-treated mice (Figure 4B), suggesting the potential of boldine in extending the life span of mutSOD1 mice. However, the difference in survival curve did not reach statistical significance (*p* = 0.0516) possibly due to limited number of subjects. We have also noticed that some mice treated with 100 mg/kg/day boldine show signs of extended survival, but the median survival for this high dose treatment group is only 120 days. Thus, the higher dose seemed to be less effective than the 50 mg/kg/day dose or might be similar to the median found with 50 mg/kg/day and the recorded difference reflects experimental variations.

### Boldine slows motor function deterioration in mutSOD1 male mice

**Figure 5:**
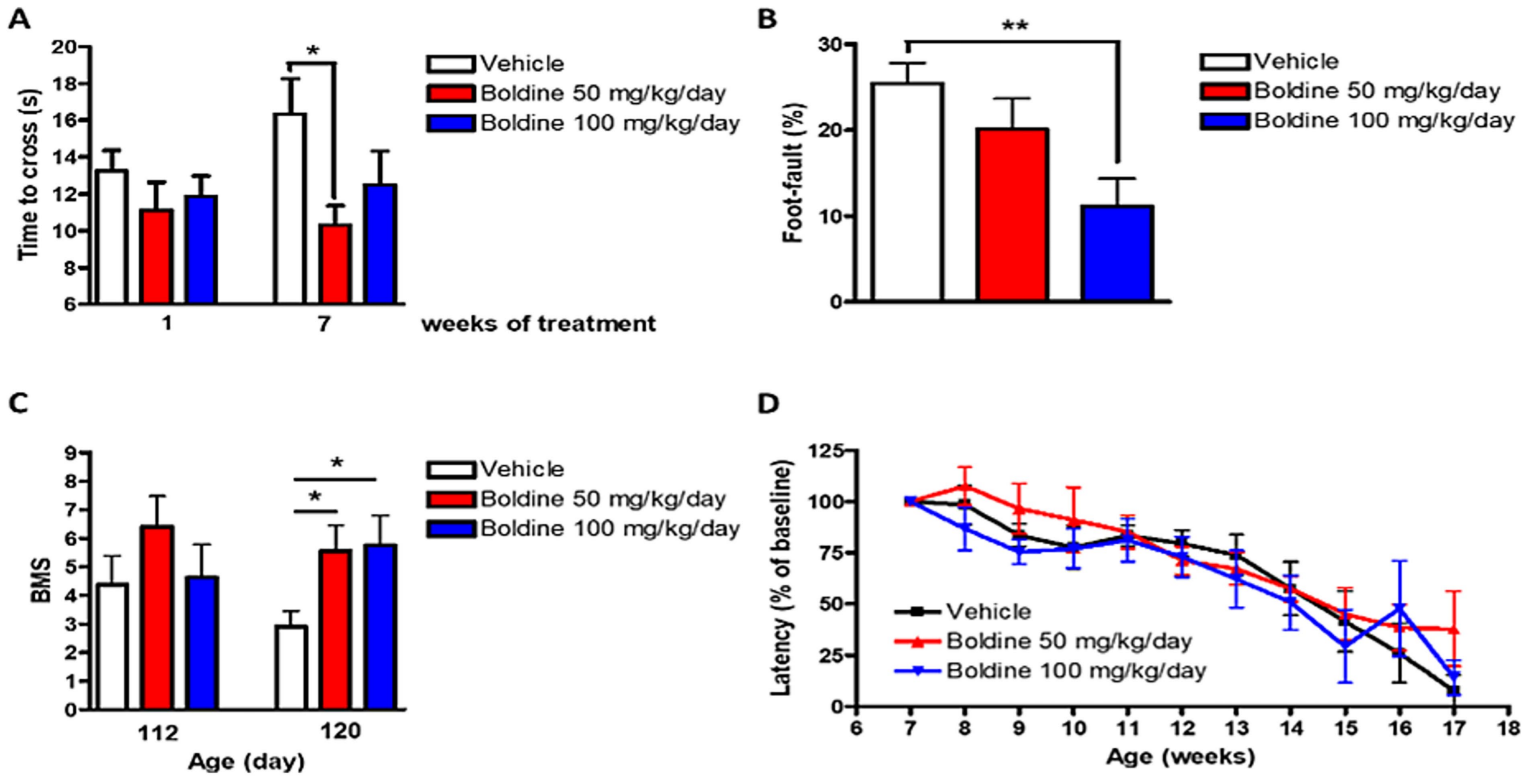
Effect of boldine on motor performance in mutSOD1 mice. Male mutSOD1 mice were fed with vehicle, 50 mg/kg BW/day or 100 mg/kg BW/day boldine (*n* = 8 per group) starting from 7 weeks of age. Horizontal ladder rung walk test (LRWT) was performed post treatment. **(A)** Time to cross the ladder and **(B)** Percentage of stepping errors were evaluated; **(C)** BMS score at 112 and 120 days of age; **(D)** Maximal motor performance was assessed by accelerating rotarod. Latency on the rotarod was expressed as percentage of baseline. Data are shown as mean ± SEM. *p < 0.05, **p < 0.01 by student *t*-test, *N* = 8 per group.

The horizontal ladder rung walk test (LRWT) (Cummings et al., 2007) for fine motor skills and coordination was performed weekly post vehicle or boldine treatment as described in our previously published studies (Toro et al., 2021). At 7 weeks post treatment, mutSOD1 mice treated with 50 mg/kg/day boldine required significantly less time to cross the ladder (−36.8% vs. vehicle-treated mice, *p* < 0.05 by *t*-test) (Figure 5A). Stepping errors were counted and expressed as percent of total steps. While vehicle treated animals showed 25.5% of stepping errors, boldine treated mice showed 20.1% (boldine 50 mg/kg/day) and 11.1% (boldine 100 mg/kg, *p* < 0.01 by *t*-test) of stepping errors (Figure 5B). Mice were also scored using the Basso Mouse Scale (BMS) (Basso et al., 2006) after disease onset. At 110 days, mice in the 50 mg/kg/day group tended to have higher BMS scores although this difference was not significant. At the age of 120 days, both boldine-treated groups showed significantly higher BMS score (Figure 5C, *p* < 0.05 by *t*-test). An accelerating rotarod was used to assess the maximal motor performance in ALS mice as described in our previous publications (Zhao et al., 2012; Zhao et al., 2011). No differences were detected in vehicle- or boldine-treated mice (Figure 5D). The above locomotor test data suggest that despite not showing a beneficial effect on maximal motor performance, boldine significantly improves fine motor performance and coordination in the mutSOD1 mouse model of ALS.

### *In silico* binding of boldine to Cx43 HC

It has been reported that Cx HC could mediate neuroinflammation in multiple disease conditions (Leybaert et al., 2017). We have previously shown that boldine blocks Cx HC in multiple model systems including mouse hippocampal slices and MES-13 mesangial cells (Hernández-Salinas et al., 2013; Yi et al., 2017); we have shown that boldine blocks open Cx26 and Cx30 HC expressed in HeLa cells which otherwise lack Cx (Toro et al., 2023). Previously, we reported that the compound D4 mitigates neuroinflammation by inhibiting Cx43 HCs. Molecular structural analysis revealed that D4 binds to a specific domain within the Cx43 HCs (Cisterna et al., 2020). This domain includes the N-terminal helix (NTH), transmembrane helix 1 (TMH1), and TMH2 of one protomer, alongside TMH1 from an adjacent protomer located beneath the NTH (Li et al., 2023; Guo et al., 2022). To begin to understand whether boldine binds Cx43 HC, this same molecular dynamics simulation was repeated. Our findings revealed that the binding interactions for boldine mirrored those observed for D4, suggesting that boldine may also anchor within a domain encompassed by the NTH, TMH1, and TMH2 across two protomers (Supplementary Figure 3A). Notably, a hydrophobic pocket appears to facilitate the binding of boldine through *π*-stacking, cation-π, and van der Waals forces, engaging with Phe97 in TMH2, Trp4 in the NTH, Val28 in TMH1, and Leu93 in TMH2 (Supplementary Figures 3B,C). Using the MM-GBSA approach, we observed a considerable binding energy of −62.7 ± 2.9 kcal/mol. The main contributors of this energy were lipophilic interactions and van der Waals forces. To confirm these findings, we evaluated effects of boldine on permeability of Cx43 HC expressed in HeLa cells (Supplementary Figure 4). Cells were incubated for 20 min in saline solution (HBSS buffer) supplemented with (dye) with Ca^2+^ (negative control) or without Ca^2+^ (which opens Cx HC) and dye uptake was determined by image analysis of live cell images. As expected, for cells maintained in media containing Ca^2+^, very few HeLa cells expressing Cx43 were labeled with dye, whereas many cells were labeled in Ca^2+^-free media. Importantly, dye uptake in Ca^2+^-free saline solution was almost totally prevented by addition of 50 μM boldine. The experimentally determined the IC50 for boldine in this system was 12.5 μM (Supplementary Figure 4).

## Discussion

To date, no definitive cure exists to prevent or halt the progression of ALS. The FDA has approved only two palliative treatments: riluzole, a glutamatergic neurotransmission and voltage-dependent sodium channel blocker (Bensimon et al., 1994; Fritz et al., 2013; Doble, 1996; van Zundert et al., 2008; Bellingham, 2011), and edaravone, an antioxidant with an unclear molecular mechanism (Cruz, 2018). The multifactorial cellular etiology of ALS continues to challenge effective drug development (Ilieva et al., 2009; Van Harten et al., 2021; Stoklund Dittlau and Van Den Bosch, 2023; Garcés et al., 2024). Among the proposed pathogenic mechanisms, oxidative stress remains a prominent contributor to motor neuron death in ALS (Barber and Shaw, 2010). Furthermore, emerging evidence implicates Cx HCs in ALS progression, highlighting their blockade as a potential therapeutic strategy (Almad et al., 2016; Almad et al., 2022; Belousov et al., 2018; Takeuchi and Suzumura, 2014). Based on these findings, we investigated boldine, a naturally derived alkaloid with antioxidant properties and the ability to block Cx HC activity (Hernández-Salinas et al., 2013; Yi et al., 2017), as a candidate therapeutic for ALS.

### Hemichannel blockade by boldine

Pathological conditions such as ischemia (Contreras et al., 2002), oxidative stress (Ramachandran et al., 2007), Duchenne muscular dystrophy (Cea et al., 2016), Alzheimer’s disease (Orellana et al., 2011), and ALS (Almad et al., 2016; Almad et al., 2022) are associated with increased Cx HC activity. In addition, Panx1 HC activity is also increased in neurons under ischemia (Thompson et al., 2006). All these conditions are characterized by increased generation of ROS, which heightens the activity of Cx HCs (Retamal et al., 2006; Figueroa et al., 2013). In the same line of thought, our study demonstrated elevated Cx HC activity in motor neurons exposed to conditioned media from mutSOD1 astrocytes. Treatment with boldine effectively reduced this activity, producing effects comparable to those of the known Cx HC blocker La^3+^ (Contreras et al., 2002; Anselmi et al., 2008). These results align with prior studies indicating that Cx HC-mediated communication exacerbates neuroinflammation and neuronal dysfunction in ALS (Almad et al., 2016; Cui et al., 2014; Almad et al., 2022; Takeuchi and Suzumura, 2014).

Furthermore, ATP release has been observed in other neurodegenerative diseases and is considered an indicator of Cx43 HC activity (Orellana et al., 2011). Studies have assessed Cx43 HC activity by measuring ATP release via a luciferase assay in the supernatants of human induced pluripotent stem-cell-derived astrocytes (hiPSC-A) from both (fALS) and sALS patients (Almad et al., 2022). At baseline, ATP concentrations in the supernatants of both fALS and sALS hiPSC-A were higher compared to controls. However, treatment with Gap19, a Cx43 HC-specific mimetic peptide blocker (Abudara et al., 2014), did not alter ATP levels in control samples but reduced ATP concentrations in fALS and sALS hiPSC-A supernatants to control levels. These findings suggest that ALS hiPSC-A exhibit increased baseline HC permeability to ATP, which can be pharmacologically inhibited.

In addition to small molecules and peptide-based inhibitors, a recent study has demonstrated that a monoclonal antibody targeting Cx43 can selectively block its hemichannel activity without interfering with gap junction communication. This antibody was shown to reduce neuroinflammatory signaling and improve neural function in preclinical models, highlighting a novel immunotherapeutic approach to modulate connexin-related pathology. Such findings reinforce the growing body of evidence supporting Cx43 as a viable drug target in ALS and related neurodegenerative disorders. When combined with our findings on boldine, these results underscore the potential of Cx HC inhibition—via pharmacological or biologic modalities—as a central strategy to mitigate astrocyte-mediated neurotoxicity in ALS. The fluorescence intensity analysis of Cx43 immunostaining in mutSOD1 astrocytes are consistent with prior findings (Almad et al., 2016; Almad et al., 2022; Bautista et al., 2014). We recognize a limitation of the present work is the lack of multiple biological replicates to further validate these observations.

### *In silico* modeling of boldine-Cx43 interaction

Molecular dynamics simulations predicted that boldine bound Cx43 hemichannels and suggest that boldine may inhibit Cx43 HC function by physically occluding a conformationally sensitive region involved in channel gating. Studies with HeLa cells transfected with plasmids expressing Cx43 demonstrated that boldine prevented dye uptake under conditions that open Cx HC consistent with prior studies which concluded that boldine blocks astrocytic and microglial connexins in culture and tissue slices and comprise Cx43 and to a small extent Cx30 (Toro et al., 2023; Yi et al., 2017). These complementary findings in a heterologous system further strengthen the mechanistic interpretation of our astrocyte-based data and reinforce the conclusion that boldine directly inhibits Cx43 HC activity.

### Antioxidant properties of boldine

Oxidative stress is a critical driver of neurodegeneration in ALS and other neurodegenerative diseases (Sanders et al., 2014; Shi and Gibson, 2007). In our study, exposure to mutSOD1 astrocyte-conditioned media led to a marked increase in ROS/RNS, contributing to motor neuron death. Boldine significantly reduced ROS/RNS levels, supporting its role as a potent antioxidant. Its radical-scavenging activity, mediated by its tertiary nitrogen atom (O’Brien et al., 2006), may help mitigate oxidative damage. Alternatively, Cx HC blockade prevents the activation of several Ca^2+^-dependent intracellular metabolic pathways that generate ROS (Balboa et al., 2020). Similarly, other antioxidants, such as vitamin E and resveratrol, also act as inhibitors of Cx43 HCs (Balboa et al., 2020), which are permeable to Ca^2+^ (Schalper et al., 2010). Thus, the antioxidant effect of boldine in inflamed cells can be complemented by its inhibitory effect of Cx HC activity by preventing the activation of Ca^2+^-dependent metabolic pathways, providing a dual mechanism to counteract ALS-related neurodegeneration.

### Neuroprotective effects of boldine

Motor neuron vulnerability in ALS arises from mechanisms including glutamate excitotoxicity, mitochondrial dysfunction, and endoplasmic reticulum stress, hyperexcitability (Garcés et al., 2024; van Zundert et al., 2012; Howland et al., 2002; Nishitoh et al., 2008; Damiano et al., 2006). In our *in vitro* ALS model, exposure to mutSOD1-conditioned media reduced motor neuron survival by approximately 50%. Boldine treatment not only prevented survival to near-control levels but also reduced both Cx HC activity and ROS/RNS. These findings demonstrate boldine’s potential to directly counteract key pathogenic mechanisms in ALS and validate its dual role as a neuroprotective agent.

Since cell death is the outcome of strong neuroinflammation in brain regions without regeneration capacity, the reduction of neuronal death described herein, could be explained by the anti-inflammatory effect boldine, which would be similar to protective effect of Cx HC inhibition found in animal models of epilepsy, depression, multiple sclerosis, and Alzheimer’s disease (Yi et al., 2017; Li et al., 2023; Guo et al., 2022; Li et al., 2020). In all these disease model the inhibition of Cx HC drastically reduces the neuroinflammation and neuronal suffering.

### Translational and future considerations

While our *in vitro* findings highlight boldine’s therapeutic promise, *in vivo* studies reveal important nuances. Boldine slowed fine motor function deterioration in male mutSOD1 mice treated with 50 mg/kg/day. However, variability in survival outcomes, particularly at higher doses, underscores the need for further optimization of dosing and delivery strategies. Future studies will investigate sex-specific effects using harmonized protocols, which merits a deeper investigation.

Notably, boldine’s ability to cross the blood–brain barrier (Loghin et al., 2003) is encouraging, but methods such as nanoparticle-based delivery could enhance bioavailability and reduce variability. Additionally, potential long-term hepatic toxicity must be addressed to ensure clinical safety (Neuman et al., 2015).

We acknowledge that the current study does not include a new Western blot analysis for Cx43, a decision made because identical experimental conditions and results have been previously published (Bautista et al., 2014). Nonetheless, we recognize this as a limitation and plan to include additional protein-level validation in future studies.

### Broader implications of boldine

Beyond ALS, boldine has demonstrated efficacy in other pathological contexts, including diabetes, osteoporosis, epilepsy and neurodegeneration (Hernández-Salinas et al., 2013; Yi et al., 2017; Moezi et al., 2019; Sasaguri et al., 2017). Its ability to modulate oxidative stress and inhibit Cx HC activity suggests potential utility across a wide range of diseases that present neuroinflammation. However, detailed pharmacokinetic studies and safety assessments are essential before advancing boldine to clinical trials.

Our findings underscore boldine’s therapeutic potential in ALS, demonstrating its ability to inhibit Cx HC activity, reduce oxidative stress, and promote motor neuron survival. These results provide a strong foundation for further investigation into boldine’s molecular mechanisms and optimization for clinical use. If validated, boldine could represent a novel therapeutic strategy for ALS and other neurodegenerative diseases characterized by oxidative stress and aberrant Cx HC activity.

## Data Availability

The raw data supporting the conclusions of this article will be made available by the authors, without undue reservation.
