# Supplementary material for: Boldine as a neuroprotective agent against motor neuron degeneration in models of amyotrophic lateral sclerosis
Source: Front Cell Neurosci. 2025 Sep 16;19:1640590. doi: 10.3389/fncel.2025.1640590 (PMC12481613; doi:10.3389/fncel.2025.1640590)
Supplement: Supplementary file 1 [file Data_Sheet_1.PDF]

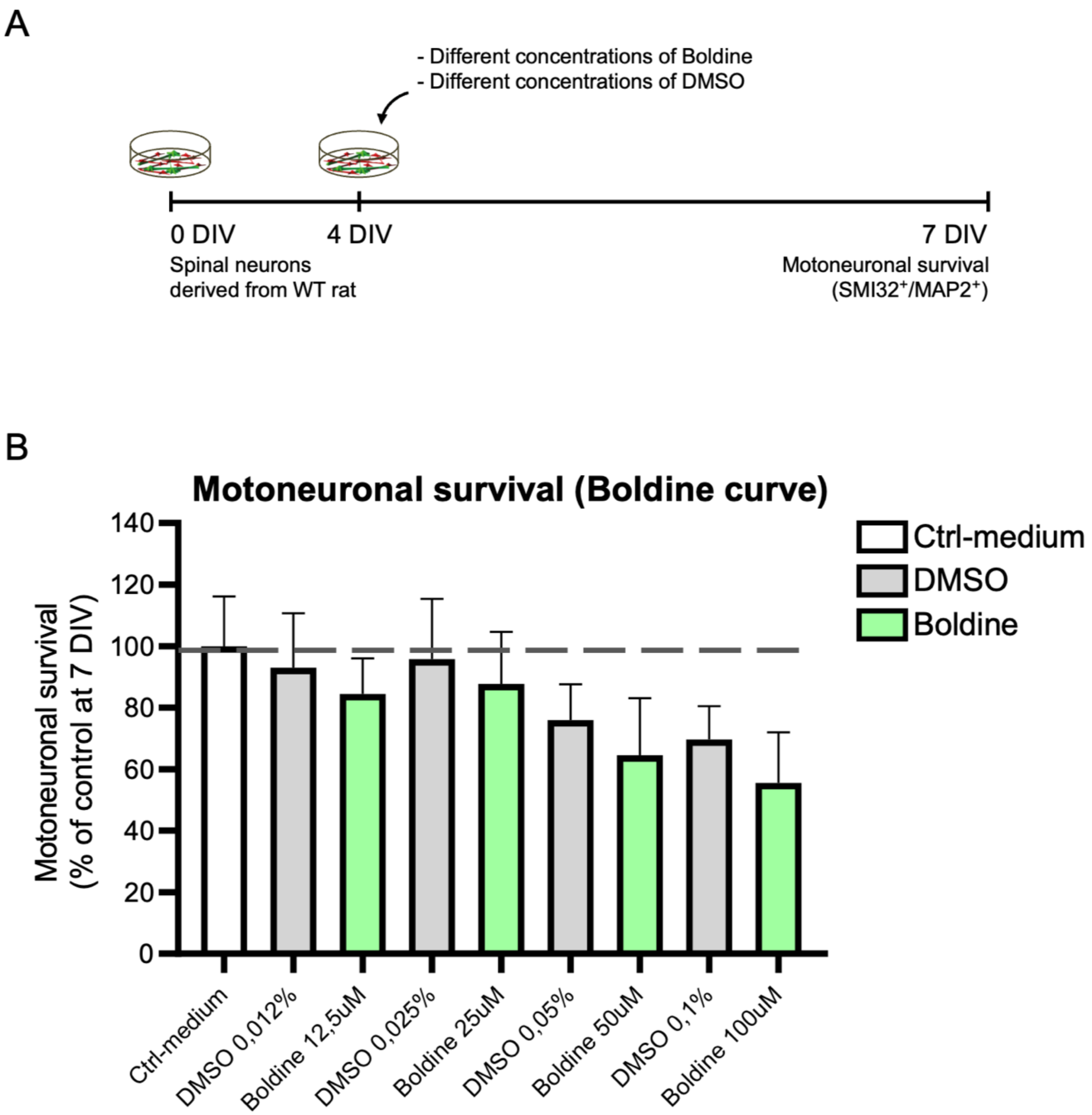

Supplementary Figure 1: Boldine at 25 μM is the highest drug concentration with no cytotoxic effect on motor neurons in spinal cord cultures. (A) Experimental design: 4 DIV spinal cord cultures were exposed to different boldine concentrations (100, 50, 25, and 12.5 μM) and DMSO (0.1, 0.05, 0.025, and 0.0125%), or control medium (Ctrl-medium), and incubated at 37°C with 5% CO_2 for 3 days. (B) Bar graph showing the survival percentage of motor neurons in each experimental condition. Values represent the mean ± standard error of at least three independent experiments.

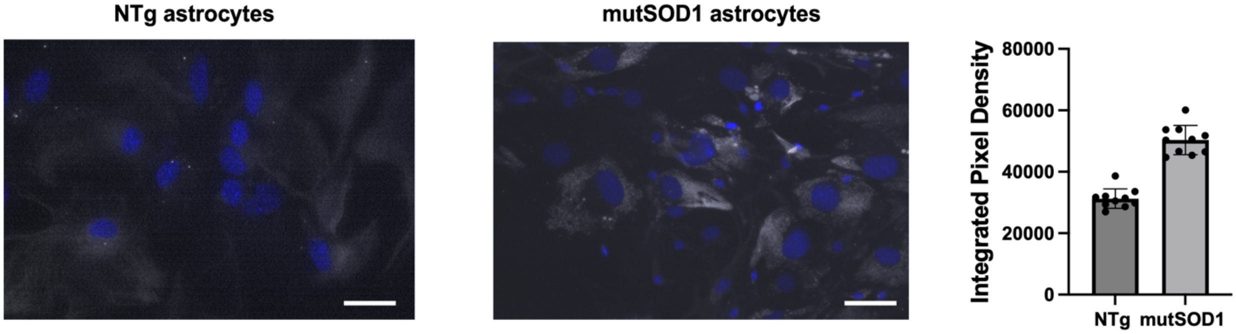

Supplementary Figure 2: Cx43 expression in control NTg and mutSOD1 mouse primary spinal cord astrocytes. Representative epifluorescence microscopy images of control NTg (left) and mutSOD1 (right) astrocytes immunostained for Cx43 (grey). Cell nuclei were stained with DAPI (blue). Quantification indicate that the average fluorescence intensity obtained with Cx43 is 50341 ± 4544 arbitrary units (AU; mean ± SD) in mutSOD1 astrocytes compared to 31261 ± 3025 AU in NTg controls (n = 1). Scale bar 25 μm.

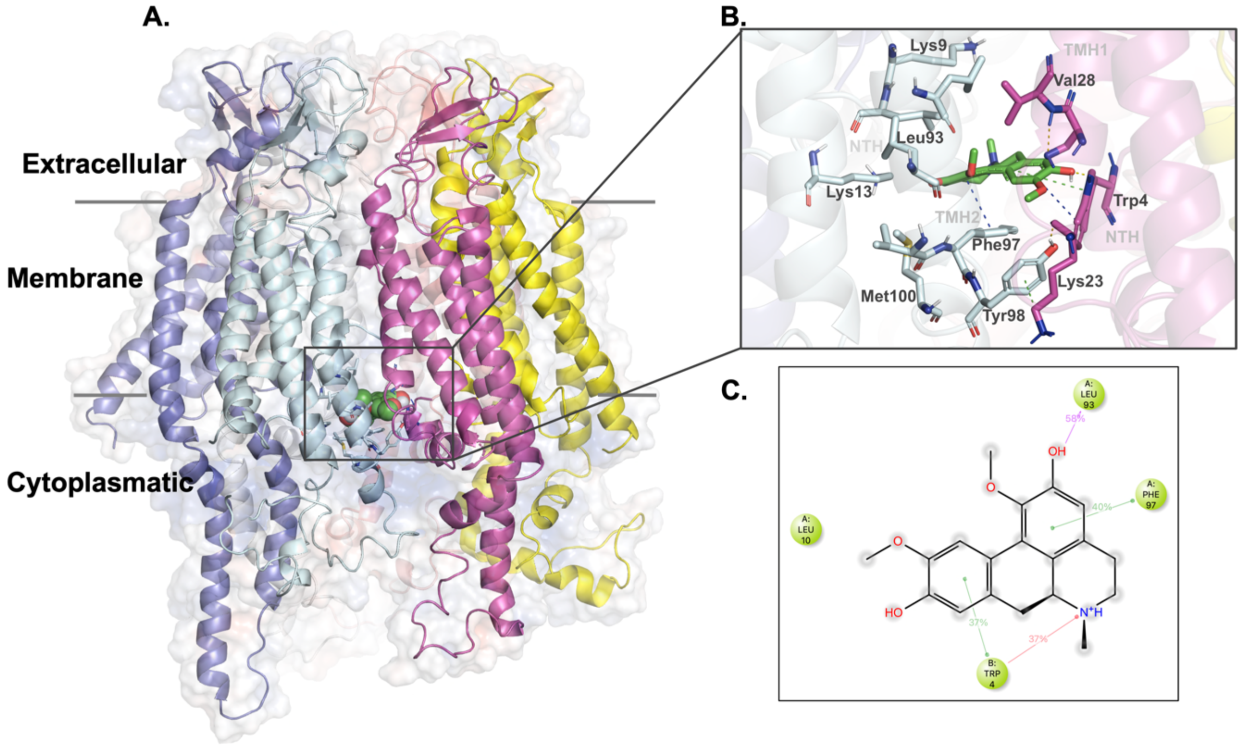

Supplementary Figure 3. Molecular docking of boldine to Cx43 hemichannel. (A) A complete structure of Cx43 hemichannel reveals the boldine binding site (highlighted in green); (B) Boldine (highlighted in green) is positioned centrally between the NTH, TMH1, and TMH2 areas. The picture shows two protomers of the connexin-43 hemichannel (highlighted in fuchsia and light blue, respectively); (C) The intricate interaction between boldine and molecular dynamics simulation. Licorice style highlights the nearest amino acids from both protomers.

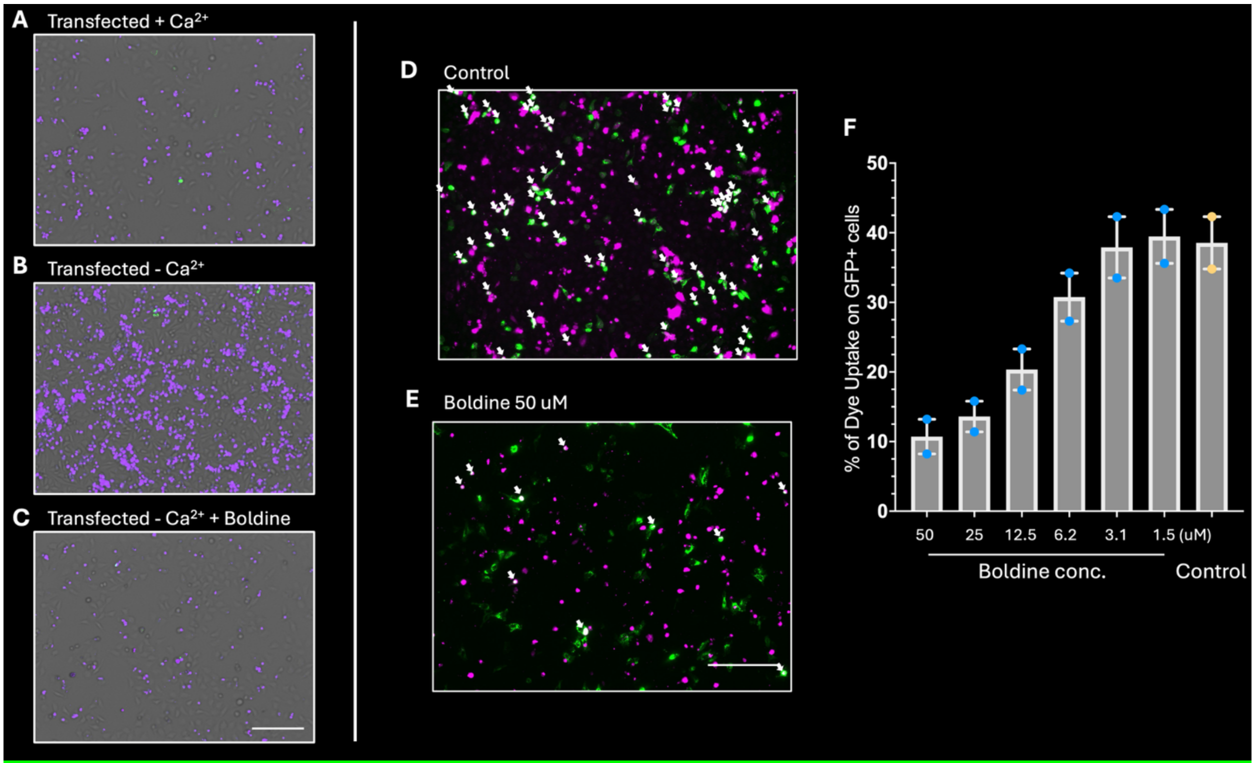

Supplementary Figure 4. Boldine inhibits TO-PRO-3 dye uptake in HeLa cells expressing Cx43-GFP. (A - C) Representative images of HeLa cells transfected with human Cx43-GFP and incubated with TO-PRO-3 dye (magenta). (A) Cells were washed with HBSS buffer containing Ca^{2+} and then incubated with the dye for 20 minutes (negative control). (B) Cells were washed with Ca^{2+}-free HBSS buffer and incubated with the dye for 20 minutes, resulting in increased dye uptake due to hemichannel activity. (C) Cells were pre-incubated with boldine (50 µM) for 2 minutes prior to dye addition, showing reduced dye uptake. (D – E) Merged fluorescence images of HeLa cells transfected with human Cx43-GFP (green) and incubated with TO-PRO-3 dye (magenta). (D) Cells were washed with Ca^{2+}-free HBSS buffer and then incubated with the dye for 20 minutes exhibited robust dye uptake. (E) Pre-treatment with boldine (50 µM) for 2 minutes prior to dye addition markedly reduced dye uptake. (F) Concentration-response curve showing TO-PRO-3 dye uptake at 20 minutes across decreasing concentrations of boldine. Images were acquired using a 10x objective. N = 2 independent experiments.
